# Case report: Changes in the levels of stress hormones during Takotsubo syndrome

**DOI:** 10.3389/fcvm.2022.931054

**Published:** 2022-07-22

**Authors:** Pablo Ruiz, Paul Gabarre, Camille Chenevier-Gobeaux, Hélène François, Mathieu Kerneis, John A. Cidlowski, Robert H. Oakley, Guillaume Lefèvre, Mathieu Boissan

**Affiliations:** ^1^Assistance Publique-Hôpitaux de Paris (AP-HP), Hôpital Tenon, Laboratoire de Biochimie, Paris, France; ^2^Assistance Publique-Hôpitaux de Paris (AP-HP), Hôpital Tenon, Soins Intensifs Néphrologiques et Rein Aigu (SINRA), Paris, France; ^3^Assistance Publique-Hôpitaux de Paris (AP-HP)-Centre Université de Paris, Hôpital Cochin, Department of Automated Biological Diagnostic, Paris, France; ^4^Sorbonne Université, Inserm, UMR_S1155, Paris, France; ^5^Sorbonne Université, ACTION Study Group, INSERM UMRS_1166, Institut de Cardiologie (AP-HP), Paris, France; ^6^Signal Transduction Laboratory, National Institute of Environmental Health Sciences, National Institutes of Health, Research Triangle Park, NC, United States; ^7^Sorbonne Université, Inserm, Centre de Recherche Saint-Antoine (CRSA), Paris, France

**Keywords:** cortisol, copeptin, cardiac biomarkers, Takotsubo syndrome, case report

## Abstract

**Background:**

Takotsubo syndrome is an acute cardiac condition usually involving abnormal regional left ventricular wall motion and impaired left ventricular contractility. It is due mainly to hyper-stimulation of the sympathetic nerve system, inducing an excess of catecholamines, usually triggered by intense psychological or physiological stress. The relationship between Takotsubo syndrome and the circulating stress hormones cortisol and copeptin (a surrogate marker of arginine vasopressin) has not been well documented.

**Case summary:**

Here, we describe the dynamic changes in circulating cortisol and copeptin during an entire episode of Takotsubo syndrome in a post-partum woman after spontaneous vaginal delivery. The patient was diagnosed with inverted Takotsubo syndrome accompanied by HELLP syndrome. We found qualitative and quantitative changes in cortisol: a loss of circadian rhythm and a three-fold elevation in the plasma concentration of the hormone with a peak appearing several hours before circulating cardiac biomarkers began to rise. By contrast, levels of copeptin remained normal during the entire episode.

**Discussion:**

Our findings indicate that the levels of cortisol change during Takotsubo syndrome whereas those of copeptin do not. This association between elevated cortisol and Takotsubo syndrome suggests that aberrant levels of this stress hormone may contribute to the observed cardiac pathology. We conclude that biochemical assays of circulating cortisol and cardiac biomarkers may be a useful complement to the diagnosis of Takotsubo syndrome by non-invasive cardiac imaging.

## Introduction

Takotsubo syndrome is a type of acute heart failure that usually involves apical ballooning of the left ventricle but can also involve the right ventricle (1). This syndrome mainly affects post-menopausal women. It resembles acute coronary syndrome but usually occurs in the absence of obstructive coronary artery disease. One of the most important triggers of Takotsubo syndrome is intense psychological or physiological stress (2), which is generally considered a hallmark of the disease. Stress-induced hyperactivation of the sympathetic nervous system plays a central role in the pathophysiology by inducing excess catecholamines. Most documented cases of Takotsubo syndrome have been linked to a recognizable emotional or physiological trigger and excess catecholamine release (3–6).

The hypothalamic-pituitary-adrenal axis is the major neuroendocrine system that responds to psychological and physiological stress (7). The major stress hormones released by this system that are monitored in clinical practice are cortisol and arginine vasopressin. Although Takotsubo syndrome is a stress-induced cardiac disease, its effects on circulating cortisol and copeptin, a surrogate marker of arginine vasopressin, have not been well documented and have never been correlated with changes in cardiac biomarkers over the entire course of an episode of Takotsubo syndrome.

Here, we report a comprehensive analysis of the changes in levels of circulating stress hormones and cardiac biomarkers in a woman presenting with an unusual case of Takotsubo syndrome soon after spontaneous vaginal delivery of her baby. We found that the two stress hormones responded differently: the amount of cortisol peaked prior to the onset of cardiac necrosis, as indicated by increased levels of circulating troponin I and troponin T, while the amount of copeptin remained normal throughout the entire episode. This suggests that cortisol may be involved in the cardiac damage observed during Takotsubo syndrome and that this hormone may be a useful addition to the panel of markers used to confirm the initial diagnosis of this disease by cardiac imaging.

## Methods

Levels of copeptin and cortisol in plasma samples collected in lithium heparin were assayed by using the KRYPTOR™ (ThermoFisher Scientific, Asnières sur Seine, France) and the Architect *ci 8200 (*Abbott, Rungis, France) analyzers, respectively. Levels of copeptin (B·R·A·H·M·S™ Copeptin proAVP) were measured by using the TRACE technology and levels of cortisol (ARCHITECT Cortisol Reagent Kit) were measured using a mouse monoclonal antibody and a competitive one-step chemiluminescence method. Normal values of copeptin and cortisol (08:00 h) were <10 pmol/L and <550 nmol/L, respectively (manufacturer's data). Internal quality controls showed a precision of <6 % at 5 pmol/L and 100 pmol/L for copeptin and <9% at concentrations below 500 nmol/L for cortisol.

Levels of cardiac high-sensitivity troponin I (cTnIhs) and troponin T (cTnThs) in plasma samples collected in lithium heparin were assayed by using the Architect *ci 8200 (*Abbott, Rungis, France) and Cobas E801 (Roche Diagnostics, Grenoble, France) analyzers, respectively. Both assays used specific antibodies for each cardiac isoform detected by chemiluminescence. Normal values of cTnIhs and cTnThs (women 99^th^ percentile) were <15.6 ng/L and <9 ng/L, respectively (manufacturer's data). Internal quality controls showed a precision of <10% at 35 ng/L for cTnIhs and a precision of <10% at 13 ng/L for cTnThs. Plasma levels of N-terminal prohormone of brain natriuretic peptide (NT-proBNP) were assayed by a sandwich assay with ElectroChemiLuminescence (ECL) technology adapted to the Cobas E801 analyzer. Normal values of NT-proBNP (women 97.5^th^ percentile) were <254 ng/L. Internal quality controls showed a precision of 4 % at 200 pmol/L and 850 pmol/L for NT-proBNP. Routine biochemical parameters in the blood samples were assayed by using the Architect *ci 8200* analyzer *(*Abbott, Rungis, France). Average values of these parameters over several days were presented as mean ± SD and compared to reference values (Z-test).

## Case presentation

The details of this case were presented in a previous report (8). In brief, a 38-year-old woman (gravida 2, para 0) was admitted to the delivery unit after a normal pregnancy with no pregnancy-induced hypertension. She was in a good mood when she arrived in the labor room. She explained that this pregnancy was very important for her because of prior difficulty falling pregnant. Because of this difficulty, she had adopted a child the year before. She reported no significant medical history other than endometriosis that was responsible for an early miscarriage a few years ago. Physical examination and biochemical results at admission were normal and she delivered a healthy baby with no complications. However, just after the delivery, she became psychologically very stressed. She did not explain the reason for her distress. She only reported that a midwife acted unkindly just after the delivery.

Thirty hours after delivery, the patient complained of a strong headache accompanied by epigastric pain. Cardio-pulmonary and neurological examination was normal, but biochemical analyses indicated HELLP syndrome and acute renal failure associated with proteinuria, microangiopathic signs (thrombocytopenia, decrease of haptoglobin concentration) and hepatic cytolysis. The patient was admitted to the nephrology intensive care unit where, in addition to the typical HELLP syndrome, we found substantial elevation of the cardiac troponin I marker cTnIhs. A twelve-derivation electrocardiogram demonstrated no pathological features, but non-invasive cardiac imaging by coronary computed tomographic angiography, transthoracic echocardiography, and cardiovascular magnetic resonance imaging revealed inverted Takotsubo syndrome, a rare variant of this disease that presents with basal ballooning instead of apical ballooning.

**Figure 1:**
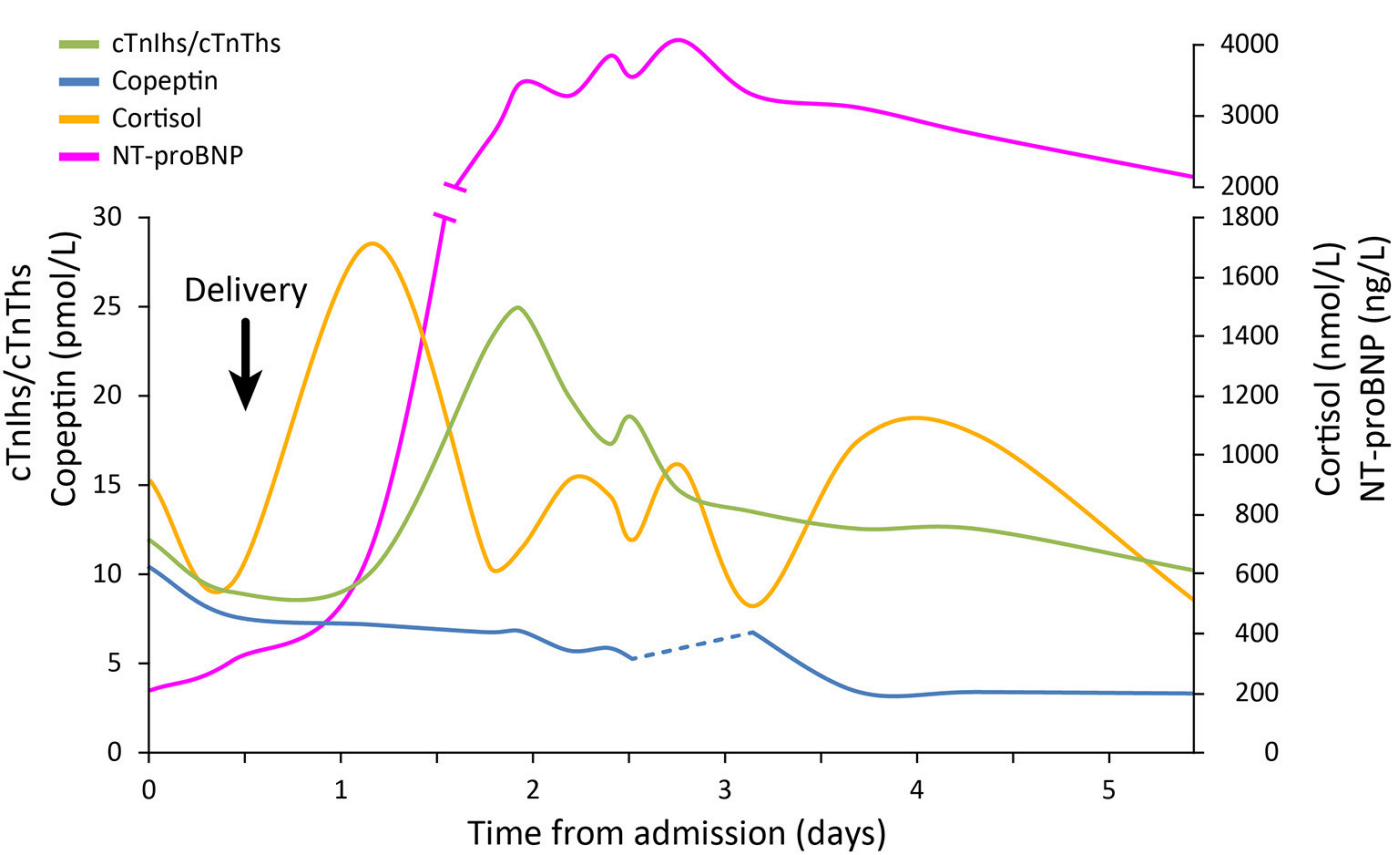
Changes in stress hormones and cardiac biomarkers during Takotsubo syndrome. Concentrations of circulating stress hormones and cardiac biomarkers were measured upon admission to hospital (day 0) and at intervals until day 5. The patient delivered her baby between day 0 and day 1 after admission. Ratio of cTnIhs/cTnThs was calculated from individual results expressed in ng/L.

Analysis of blood stress hormones in conserved plasma samples showed that copeptin levels remained normal throughout the entire episode of Takotsubo syndrome i.e. before and after the biological signs of cardiac involvement. The highest level of copeptin (10.5 pmol/L) was observed at admission, before delivery, but this was within the normal range, and the lowest level of copeptin (3.3 pmol/L) was observed at day 3. By contrast, the circadian rhythm of cortisol levels was probably affected from day 1 to day 3 since morning and evening concentrations were similar; for example, on day 2, cortisol was 920 nmol/L in the morning and 966 nmol/L in the evening. The normal rhythm was re-established on day 4. Quantitatively, the levels of circulating cortisol strongly increased between admission and day 1 peaking at 1,714 nmol/L very early in the morning of day 1. This elevation corresponded to a >3-fold increase in the maximal cortisol concentration usually observed at 08:00 h in healthy individuals (550 nmol/L). The peak of cortisol was observed several hours before the levels of circulating cardiac biomarkers began to increase (Figure 1). Levels of cTnIhs and cTnThs, indicative of cardiac necrosis, reached a peak 19 h after the peak of cortisol, with a ratio cTnIhs/cTnThs always >1, together with a peak in the level of natriuretic peptide NT-proBNP, indicative of cardiac stretching. Plasma concentrations of electrolytes (sodium, chloride, bicarbonates), urea, creatinine, total proteins, calcium, magnesium, and phosphorus remained within the normal ranges during the entire episode of Takotsubo syndrome from admission to day 5 (expressed as means ± SD: sodium, 137.8 ± 1.5 mmol/L; chloride, 104.6 ± 1.4 mmol/L; bicarbonates, 22.8 ± 1.7 mmol/L; urea, 4.1 ± 0.8 mmol/L; creatinine, 74.6 ± 10.9 μmol/L; total proteins, 67.9 ± 5.6 g/L; calcium, 2.13 ± 0.20 mmol/L; magnesium, 1.21 ± 0.43 mmol/L; phosphorus, 0.89 ± 0.22 mmol/L. See Table 1). By contrast, the concentrations of potassium ranging from 3.5–4.9 mmol/L (mean ± SD, 4.2 ± 0.4 mmol/L) were significantly different when compared to normal values (*P* < 0.05). The calculated plasma osmolality (289–299 mosmol/L) was within the normal range (285–385 mosmol/L). Creatinine, a marker of renal failure when elevated, peaked 20 hours before the peak of cardiac biomarkers, but the increase of only 11%, corresponding to a small decrease in the estimated glomerular filtration rate from 72 to 63 mL/min/1.73 m^2^, was not of the same magnitude as that of cTnIhs (+ 936 %), cTnThs (+ 233%), or NT-proBNP (+ 291 %); this moderate renal injury does not explain the huge elevation of cardiac biomarkers.

**Table 1:** Changes in biochemical parameters in blood during Takotsubo syndrome.

**Date**	**Hour**	**Sodium** **(mmol/L)**	**Potassium (mmol/L)**	**Chloride** **(mmol/L)**	**Bicarbonates (mmol/L)**	**TP** **(g/L)**	**Urea (mmol/L)**	**Creat**. **(**μ**mol/L)**	**Calcium (mmol/L)**	**Phos**. **(mmol/L)**	**Mg** **(mmol/L)**
			—————————————————————— Pre-delivery —————————————————————								
Day 0	11:40 a.m.	138	4.90	106	23	58	4.7	89	N.D.	N.D.	N.D.
			——————————————————————- Post-delivery ———————————————————————-								
Day 1	05:00 a.m.	139	4.29	106	19	74	5.2	77	N.D.	N.D.	N.D.
	07:55 p.m.	136	4.40	103	23	64	4.9	88	N.D.	N.D.	N.D.
	11:55 p.m.	136	4.40	102	23	70	4.6	98	2.07	0.87	1.82
Day 2	06:00 a.m.	137	3.60	103	24	61	4.1	76	1.86	0.68	1.87
	11:00 a.m.	138	3.50	104	26	64	3.4	72	1.90	0.66	1.43
	12:25 p.m.	138	3.90	106	23	63	3.2	70	1.90	0.70	1.32
	07:50 p.m.	136	4.41	105	24	69	2.6	70	2.06	0.71	1.13
Day 3	05:00 a.m.	136	4.30	104	24	68	3.4	69	2.19	N.D.	N.D.
Day 4	06:12 a.m.	139	4.50	107	21	70	3.8	66	2.27	0.97	1.02
Day 5	09:00 a.m.	140	4.30	105	24	77	3.8	69	2.37	1.01	0.88

*TP, total proteins; Creat, creatinine; Phos, phosphorus; Mg, magnesium; N.D. not determined. Day 0 corresponds to the day of admission*.

The patient was treated with a beta-blocker (labetalol) and an angiotensin-converting enzyme inhibitor (captopril), which normalized blood pressure.

One month after discharge, transthoracic echocardiography was normal, indicating that the patient was in complete remission of her post-partum inverted Takotsubo syndrome. Three months after discharge, the patient was fully recovered, clinical examination was normal, and there was no hypertension or abnormalities in the blood analyses, thus the anti-hypertensive drugs were discontinued.

## Discussion

In this case study, we measured both the circulating stress hormones copeptin and cortisol and cardiac biomarkers throughout an entire episode of Takotsubo syndrome. We found a substantial peak in the level of cortisol that preceded by 19 h a peak in biomarkers indicative of cardiac necrosis and stretching, whereas the levels of copeptin remained normal throughout. As stress is an important trigger of this syndrome and is generally considered a hallmark of the disease, our finding provides a biological correlate of stress that might be important for the pathophysiology of the syndrome and might be used to confirm the initial diagnosis of this disease by cardiac imaging.

Changes in stress hormones other than catecholamines in Takotsubo syndrome are very poorly documented in the literature. Whereas most reported studies measured stress hormones only at single timepoint, we have analyzed them throughout an episode from before its onset to its resolution. Moreover, we have compared the changes in these stress hormones with changes in cardiac biomarkers. Finally, we have documented the levels of both copeptin and cortisol. Thus the study enriches substantially the current literature on changes in these important stress hormones during Takotsubo syndrome.

Our patient became psychologically very stressed just after the delivery, which may have contributed to triggering Takotsubo syndrome. There is a general consensus that sudden and severe emotional or physical stress is a common factor in the etiology of the syndrome, by causing a surge in catecholamines that ultimately leads to acute left ventricular dysfunction [although it was reported recently that positive emotional events can also provoke Takotsubo syndrome (9)]. The Comorbidity Frequency in the Takotsubo Syndrome (COUNTS) study found that emotional and physical stress were triggers in 39 and 35%, respectively, of 1,109 patients with Takotsubo syndrome (10). In addition, this study revealed that patients with Takotsubo syndrome had a relatively high prevalence of psychological disorders (24%). Patients with Takotsubo syndrome have also been shown to have a high prevalence of chronic anxiety disorder that preceded onset of the disease (11). Moreover, anxiety and mood disorders are predictors of Takotsubo syndrome, possibly because they are associated with a higher risk of stressful events (12). Thus, chronic psychological stress may be a risk factor for Takotsubo syndrome whereas acute stress may ultimately trigger the syndrome.

Although emotional or physical stress is the most common trigger of Takotsubo syndrome, several reports have suggested other triggers of Takotsubo syndrome including hyper- and hypothyroidism. Thyrotoxicosis is the most frequent thyroid hormone condition linked to Takotsubo syndrome. Thyroid hormones sensitize the heart to catecholamines by stimulating expression of beta-adrenoceptors in cardiomyocytes. Thyrotoxicosis might then potentiate the effects of catecholamines on the myocardium, increasing its sensitivity to stress (13).

Takotsubo syndrome is generally preceded by physical or emotional stress and the stress response is mainly mediated by activation of the hypothalamic-pituitary-adrenal axis. We expected, therefore, that both cortisol, released by the adrenal glands, and copeptin, released by the pituitary, would be elevated in our patient. Contrary to our expectations, however, the levels of copeptin remained normal during the entire episode of Takotsubo syndrome. This is consistent with the absence of change in sodium concentration and plasma osmolality, which arginine vasopressin would have modified by increasing water reabsorption and retention, thus resulting in hemodilution and dilutional hyponatremia. By contrast, we found qualitative and quantitative changes in cortisol: a likely loss of the circadian rhythm and a strong elevation in plasma concentration with a maximal value corresponding to a three-fold increase of the maximal concentration usually observed at 08:00h in normal individuals. In addition, cortisol peaked several hours before the circulating cardiac biomarkers began to increase. We cannot exclude the possibility that this elevated cortisol was due to pathophysiological stress associated with the postpartum HELLP syndrome rather than to emotional stress.

The rise in cortisol we measured in our patient with Takotsubo syndrome might be a protective mechanism to combat the adverse effects of excessive catecholamines on the heart and return it to homeostasis. Cortisol is a glucocorticoid steroid hormone. Glucocorticoids exert their actions on the heart by binding to the glucocorticoid receptor and the mineralocorticoid receptor in cardiomyocytes (14). In animals, increases in glucocorticoids and activation of signaling through the cardiomyocyte glucocorticoid receptor benefit the heart by enhancing cardiomyocyte contractility and inhibiting cardiomyocyte cell death (15–18). Conversely, deficient cardiac glucocorticoid receptor signaling leads to impaired contractility and left ventricular systolic dysfunction, which are the hallmarks of Takotsubo syndrome (19, 20). Thus, the increased cortisol in our patient might protect against these hallmarks.

**Figure 2:**
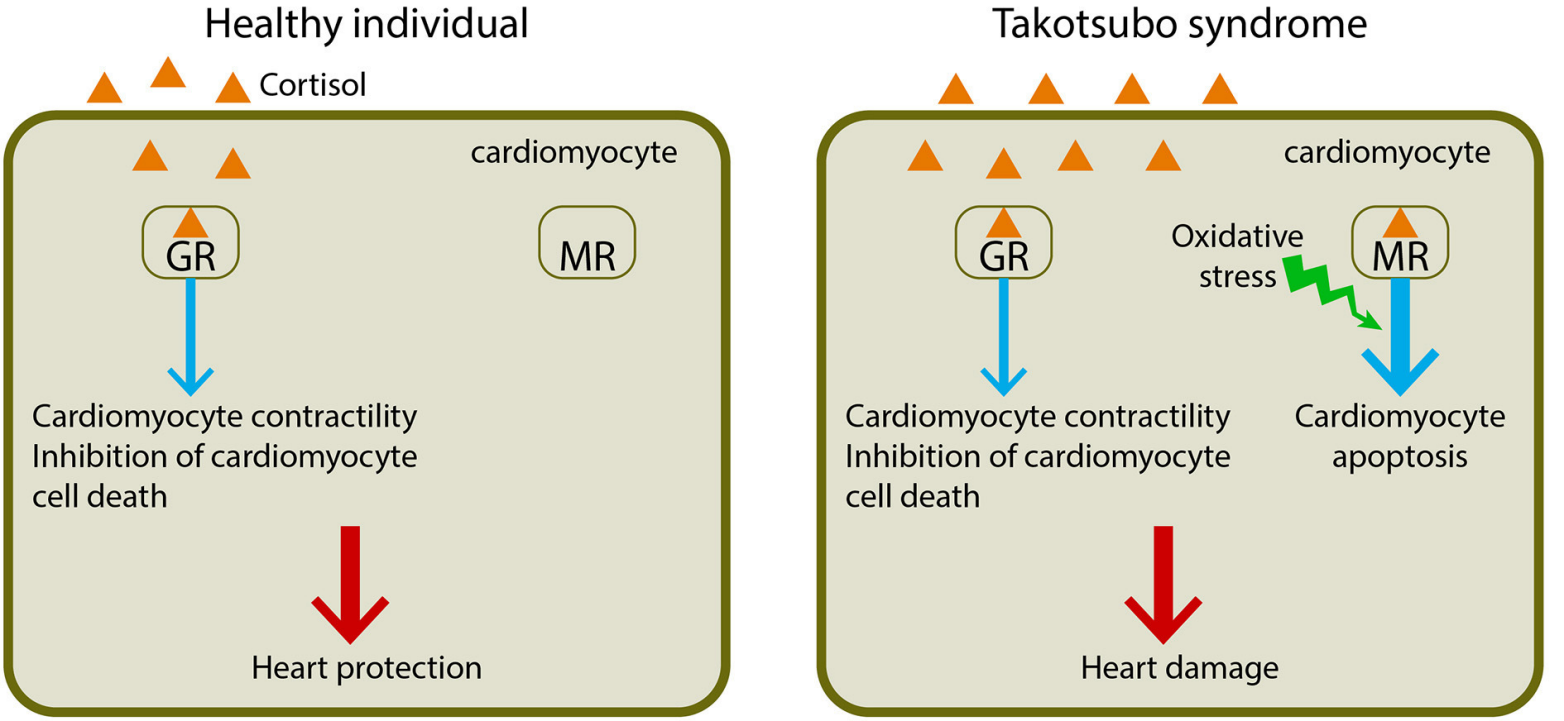
Dual effects of cortisol in cardiomyocyte function. Cortisol exerts its actions on the cardiomyocyte by binding two nuclear receptors, the glucocorticoid receptor (GR) and the mineralocorticoid receptor (MR). In a healthy individual, cortisol binds to the glucocorticoid receptor and activates its signaling, leading to enhanced cardiomyocyte contractility and inhibited cardiomyocyte death, thus protecting the heart. In a patient with Takotsubo syndrome, the increased cortisol binds not only to the glucocorticoid receptor but also to the mineralocorticoid receptor, leading to cardiomyocyte apoptosis in the presence of oxidative stress, which is often associated with Takotsubo syndrome, and thus resulting in heart damage.

An alternative interpretation, however, is that the rise in cortisol in our patient with Takotsubo syndrome exacerbates the heart pathology initially triggered by a catecholamine surge by activating signaling through the mineralocorticoid receptor (Figure 2), which promotes cardiomyocyte apoptosis and aggravates heart damage in the presence of oxidative stress (20–22). Growing evidence suggests Takotsubo syndrome is associated with increased production of reactive oxygen species, which may be involved in causing endothelial dysfunction leading to a transient impairment of myocardial contraction (23). The decreased potassium plasma levels from 4.4 to 3.6 mmol/L between day 1 and day 2 are consistent with this hypothesis and with a global activation of the mineralocorticoid signaling because activation of the mineralocorticoid receptor causes potassium excretion by the kidney. Ultimately, the severity and extent of the symptoms in Takotsubo syndrome may reflect the degree of imbalance between the positive and negative actions of cortisol on the heart.

Our finding that serum levels of copeptin were unaffected in our patient differs from a study of Takotsubo syndrome patients with atypical (midventricular) ballooning, which found significantly higher levels of copeptin than in patients with typical (apical) ballooning (24). Low copeptin levels were found, however, in two other studies of patients with Takotsubo syndrome (25, 26). Similar inconsistencies have been observed in studies of cortisol in patients with Takotsubo syndrome. In one study (27), evening plasma cortisol levels were elevated in 53% of patients with Takotsubo syndrome whereas the 24-h urine cortisol levels were normal in all patients, suggesting, like our study, that activation of the corticosteroid system is not sustained beyond the acute phase of the disease. Two other studies, however, found morning salivary cortisol levels were similar in patients with Takotsubo syndrome and in healthy individuals (28, 29).

Several factors may explain the divergent findings of our present study with others in the literature. First, the previous studies considered a single time-point, thus the transient elevation of cortisol may have been missed. The absolute amount of blood stress hormones is highly dependent on the interval between the onset of the disease (stress trigger) and blood sampling. One strength of our study is that blood samples for determination of these hormones were taken immediately after hospital admission and during all the hospital stay. Second, cortisol can be difficult to measure accurately because of its circadian rhythm and because most is bound to cortisol-binding globulin and albumin; only ~10% of total plasma cortisol is unbound and biologically active (30). Plasma cortisol assays measure total cortisol (bound and unbound fractions) and their results can be misleading in patients with altered plasma protein concentrations. In our case, the plasma protein concentration of our patient was normal. Urinary and salivary cortisol measurements reflect changes in unbound plasma cortisol, but urinary cortisol is a useful index only of integrated 24-h plasma free cortisol because it is measured in urine collected over 24 h, and salivary cortisol levels reflect only 50–70% of plasma free cortisol due to the conversion of cortisol to cortisone in saliva; measurement of salivary cortisol is not yet used in routine clinical practice. Finally, the disparate findings may be attributed to differences in the analytical assays and cut-off values for what is considered normal.

## Conclusion

Here, we describe the changes in circulating stress hormones and cardiac biomarkers during an unusual episode of Takotsubo syndrome in a post-partum woman after spontaneous vaginal delivery. Qualitative and quantitative changes in the levels of cortisol were seen: a loss of the circadian rhythm and strong elevation of the plasma concentration, with a peak several hours before the circulating cardiac biomarker levels began to rise. Levels of copeptin remained normal during the entire episode. Our findings indicate that measurement of the levels of the circulating stress hormone cortisol, in addition to cardiac biomarkers, by using biochemical assays routinely available in most non-specialized laboratories, may be a useful complement to the diagnosis of Takotsubo syndrome by non-invasive cardiac imaging. The beneficial or deleterious effects of cortisol on the heart in Takotsubo syndrome remain to be established in future studies.

## Data availability statement

The original contributions presented in the study are included in the article/supplementary material, further inquiries can be directed to the corresponding author.

## Ethics statement

Written informed consent was obtained from the individual for the publication of any potentially identifiable images or data included in this article.

## Author contributions

PR and CC-G performed biological analyses. PG, HF, and MK performed physical examination and cardiac imaging. JC, RO, GL, and MB wrote the manuscript. MB supervised the study. All authors contributed to the article and approved the submitted version.

## Conflict of interest

The authors declare that the research was conducted in the absence of any commercial or financial relationships that could be construed as a potential conflict of interest.

## Publisher's note

All claims expressed in this article are solely those of the authors and do not necessarily represent those of their affiliated organizations, or those of the publisher, the editors and the reviewers. Any product that may be evaluated in this article, or claim that may be made by its manufacturer, is not guaranteed or endorsed by the publisher.
